# Spatial Effects of Digital Transformation, PM_2.5_ Exposure, Economic Growth and Technological Innovation Nexus: PM_2.5_ Concentrations in China during 2010–2020

**DOI:** 10.3390/ijerph20032550

**Published:** 2023-01-31

**Authors:** Fenfen Ma, Shah Fahad, Mancang Wang, Abdelmohsen A. Nassani, Mohamed Haffar

**Affiliations:** 1 School of Management, Yulin University, Yulin 719000, China; 2 School of Management, Hainan University, Haikou 570228, China; 3 School of Economics and Management, Leshan Normal University, Leshan 614000, China; 4 School of Economics and Management, Northwest University, Xi’an 710127, China; 5 Department of Management, College of Business Administration, King Saud University, P.O. Box 71115, Riyadh 11587, Saudi Arabia; 6 Department of Management, Birmingham Business School, University of Birmingham, Birmingham B15 2TY, UK

**Keywords:** digital transformation, air pollution, PM_2.5_ exposure, economic development, spatial spillover effects

## Abstract

Digital transformation can increase lending by commercial banks, which may have an impact on economic development and technological progress, thus affecting air pollution. However, a limited amount of literature has discussed the impact of the digital transformation of commercial banks (DTCB) on air pollution. Based on city-level data from 2010 to 2020, this study used a spatial Durbin model to explore the spatial effects of DTCB on air pollution. This study shows that DTCB significantly increases air pollution in local and surrounding cities. Heterogeneity analysis shows that DTCB increases local and surrounding city air pollution in non-innovative cities and cities with low digital economy development. However, in innovative cities and cities with high digital economy development, DTCB reduces PM2.5 emissions in local and surrounding cities. Mechanism analysis shows that DTCB has no significant impact on technological innovation, but significantly promotes economic development, thus increasing air pollution. From the perspective of DTCB, this paper deepens the research on digital finance and air pollution. Against the background of DTCB, the government should guide commercial banks to apply digital technology to increase lending for technology innovation and promote DTCB to achieve the dual goals of economic development and improvement in air quality.

## 1. Introduction

Air pollution is harmful to the health of residents. Studies based on the experience of countries such as Greece, the United States, and China have shown that air pollution is harmful to the health of residents. Air pollution can cause respiratory and cardiovascular diseases [1], shorten life expectancy [2], and increase the infant mortality rate [3]. Therefore, reducing air pollution is of great significance for improving the health of residents.

Since air pollution is mainly generated by economic activities, many studies have investigated the impact of economic development on air quality. Grossman and Krueger (1995) found an inverted U-shaped relationship between air quality and real per capita income, and proposed the famous environmental Kuznets curve hypothesis [4]. This argues that economic development is often accompanied by technological advancement. Therefore, when economic development reaches a certain level, there is a positive correlation between economic development and air quality. In other words, although economic growth increases air pollution, technological progress can reduce air pollution [5]. The essence of finance is to serve the real economy. Financial development can promote economic growth and technological progress and thus have an impact on air pollution [6,7]. Therefore, many scholars pay attention to the effects of financial development on air pollution.

Existing studies have found that financial development positively and negatively impacts air pollution. On the positive side, financial development increases air pollution by promoting economic development [8]. Ren and Zhu (2017) call this the ‘scale effect’ of financial development [9]. The ‘scale effect’ refers to the fact that financial development effectively helps the firms expand the scale of production by alleviating their financial constraints, which stimulates energy consumption and increases air pollution [10]. On the negative side, financial development can help reduce air pollution by financing enterprises to innovate cleaner production technologies [11]. Ren and Zhu (2017) call this the ‘technology effect’ of financial development [9]. The ‘technology effect’ refers to the fact that financial development is conducive to promoting technological progress by financing of enterprise technology innovation, which reduces energy consumption per unit of output and decreases air pollution. Which indicates that, financial development has an impact on air pollution.

The current development of digital finance may affect air pollution. Digital finance refers to financial innovations enabled by technology that can create new business models, applications, processes, or products, changing financial markets, financial institutions, or the way financial services are delivered [12]. Financial institutions that apply digital finance include Fintech companies and commercial banks [13]. Financial institutions use digital technology to make breakthroughs, which are a form of financial development [14]. Cao et al. (2021) found that the development of digital finance promotes economic growth and technological innovation [15]. Huang et al. (2022) further studied whether the development of digital finance affects energy–environment performance and found that the development of digital finance has a U-shaped impact on energy–environment performance [16].

Although Huang et al. (2022) investigated the impact of digital finance development on the environment, there are still some drawbacks to this research. This study uses the Peking University-compiled digital financial inclusion index to measure the degree of development of digital finance in cities. This index can only reflect the degree of development of Fintech companies within a city. However, it cannot reflect the degree of application of digital technology by commercial banks within a city [17]. Huang et al. (2022) discussed the impact of Fintech companies on the environment, ignoring the effects of DTCB on the environment. Commercial banks are the primary source of external funding for companies. In addition, DTCB has a more significant impact on air pollution than Fintech companies. To fill this gap, the current study explores the impact of DTCB on air pollution using data of 297 Chinese cities from 2010 to 2020.

The marginal contributions of this study are as follows. First, the research object of this study is DTCB; thus, this study expands the research perspective of digital transformation. Current research focuses on exploring the effect of enterprise digital transformation, while little research has been conducted on the impact of DTCB. In contrast to enterprise digital transformation, DTCB results in economic consequences by increasing lending to enterprises, which deserves further research. Second, this study investigates the impact of DTCB on air pollution, which expands the research on the factors affecting air pollution. Scholars have discussed the impact of Fintech companies on air pollution; however, few scholars have specifically discussed the impact of DTCB on air pollution. Since the financial system of China is controlled by commercial banks, DTCB will undoubtedly have a more significant impact on air pollution. Third, the current study enriches the research on the economic consequences of DTCB. The existing literature discusses the impact of DTCB on the scale and structure of credit supply, and few studies discuss their impact on air pollution. This study further extends the research on the economic consequences of DTCB from the perspective of air pollution.

## 2. Literature Review

### DTCB Impact Mechanism and Air Pollution

1. The impact of DTCB on commercial banks’ lending

Through digital transformation, commercial banks can improve their ability to screen information and increase lending. Information asymmetry between banks and enterprises leads to credit rationing. Credit rationing refers to the insufficient supply of credit by commercial banks. The implementation of credit rationing is beneficial for commercial banks in managing credit risk under the condition of information asymmetry [18]. DTCB improves the ability to detect information in two ways. Firstly, DTCB can enhance commercial banks’ ability to collect data. Traditional commercial banks mainly collect enterprise data through manual investigation. As a result, the collected information can be subjective and can lag to a certain extent. Hence, it is difficult for commercial banks to gain a complete, accurate, and timely knowledge of enterprises. In the context of the digitalization of the economy, e-Commerce platforms, social network platforms, and credit investigation platforms have accumulated a large amount of corporate data, which can reflect company’s basic information in a more detailed way. The application of cloud computing by commercial banks can connect these data platforms and broaden information channels [19]. Commercial banks can continuously monitor enterprises using cloud computing and other network technologies, thus improving the timeliness of information acquisition. Commercial banks can also use Internet of Things (IoT) technology to track enterprise operations and output in real time. Second, the use of digital technology can boost commercial banks’ data processing capability. Traditional banks mainly conduct risk assessments on enterprises through manual analysis and a simple scoring model. Through the application of big data, cloud computing, and mathematical statistics models, commercial banks have a stronger data processing capacity [17]. By improving the ability of information screening, commercial banks can find and loan to more high-profitable enterprises, which increases lending [20].

2. The impact of increasing lending on air pollution

Lending expansion has both beneficial and bad effects on air pollution. First, increasing lending is conducive to promoting economic growth, which increases air pollution. On the supply side, increasing lending to enterprises alleviates their credit constraints and helps them expand production [21]. On the demand side, rising consumer loans enables individuals to purchase more durable consumer products (such as air conditioners and washing machines), motivating firms to boost output [22]. Expanding production certainly increases energy consumption [23,24]. In 2020, more than half of the total energy consumption came from coal. Coal is still dominating China’s energy mix. Therefore, expanding energy consumption means increasing air pollution. However, increasing lending is conducive to technological innovation, which may reduce air pollution. Increased lending to enterprises allows them to increase research and development spending, promoting technological progress. Both advanced and cleaner manufacturing methods increase output efficiency and reduce energy consumption per unit of output, reducing air pollution.

3. The impact of DTCB on air pollution

**Figure 1:**
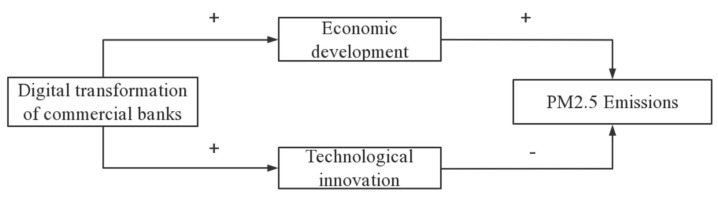
The mechanism by which DTCB affects air pollution.

In conclusion, by increasing credit supply, DTCB has both positive and negative impacts on air pollution. On the one hand, DTCB helps enterprises expand their production scale, increasing air pollution. This is called the ‘scale effect’ of DTCB [9]. On the other hand, DTCB encourages enterprise technical innovation, which reduces air pollution. This is called the ‘technology effect’ of DTCB [9]. The ‘scale effect’ of DTCB is positively correlated with air pollution, but its ‘technology effect’ is negatively correlated with air pollution. Therefore, the impact of DTCB on air pollution is uncertain. In general, the mechanism by which DTCB affects air pollution is shown in Figure 1. Therefore, the following hypotheses are proposed.

**Hypothesis** **1a.** *The ‘scale effect’ of DTCB is more significant than its ‘technology effect’, so DTCB increases air pollution.*

**Hypothesis** **1b.** *The ‘technology effect’ of DTCB is more significant than its ‘scale effect’, so DTCB reduces air pollution.*

4. Heterogeneity of innovation ability

The impact of DTCB on air pollution varies among cities with different innovation abilities. In academia, there is currently no single definition of innovation ability. Referring to the definition of innovation ability in existing studies, the innovation ability in this article refers to the ability of various subjects of innovation to organically integrate innovation factors to generate new technologies, processes, and services [25,26]. In this paper, cities are classified into innovative cities (with strong innovation capabilities) and non-innovative cities (with weak innovation capabilities). Theoretical analysis shows that DTCB decreases air pollution by driving technological innovation. However, the ‘technology effect’ of DTCB does not occur in non-innovative cities. This is because, in non-innovative cities, there is often a shortage of innovation resources, such as innovative talents. When it is difficult to find the talent they need, companies do not innovate their technology, even if they receive loans. Therefore, although DTCB can increase credit supply, it cannot promote technological innovation and thus cannot play its role in reducing air pollution. In innovative cities, there are abundant innovation resources. Companies can easily find the innovative talent they need, so they are likely to use the loans for technological innovation. In this case, DTCB can promote technological innovation by increasing loans, thus reducing PM2.5.

In conclusion, the ‘technology impact’ of DTCB is stronger than the ‘scale effect’ in creative cities, lowering air pollution. In contrast, in non-innovative cities, the ‘technology effect’ of DTCB is smaller than the ‘scale effect’, thus increasing air pollution. Therefore, the following hypothesis is proposed.

**Hypothesis** **2.** *In innovative cities, DTCB reduces air pollution. In non-innovative cities, DTCB increases air pollution.*

5. Heterogeneity of the degree of digital economy development

The impact of DTCB on air pollution varies among cities with different degrees of digital economy development. The relationship between DTCB and air pollution depends on whether its ‘technology effect’ is greater than its ‘scale effect’. In cities with high digital economy development, the ‘technology impact’ of DTCB is stronger than the ‘scale effect’. The reason for this is that, in cities with high digital economy development, DTCB can better address the financing difficulties of innovation, thereby fully enabling the ‘technology effect’ of DTCB. Innovation usually faces serious financing difficulties due to high-risk and high-level capital investment. Furthermore, to avoid revealing trade secrets, enterprises are usually reluctant to release detailed information related to their innovation projects. This exacerbates the information asymmetry in innovation financing and further undermines the willingness of commercial banks to lend for the innovation of companies [27]. In cities with high digital economy development, many enterprises within the city have completed their digital transformation [28]. If a company completes its digital transformation, its technological innovation, production and operation, internal control, and product sales will all be captured in the form of data. These data are open, transparent, shared, and verifiable, and can truly reflect the company’s innovation, production, and sales [29,30]. Commercial banks can access the above data in the credit tracking system through digital technology, effectively reducing the information asymmetry and solving the innovation financing difficulties. Therefore, in cities with high digital economy development, the ‘technology effect’ is fully enabled, and the ‘technology impact’ of DTCB may be stronger than the ‘scale effect’, thus reducing air pollution. On the contrary, in cities with low digital economy development, the ‘technology impact’ of DTCB is smaller than the ‘scale effect’. The reason for this is that, in cities with low digital economy development, DTCB can not address the financing difficulties of innovation, which makes it difficult to enable its ‘technical effect’. In cities with low digital economy development, although commercial banks can broaden the information sources through digital transformation, it is difficult to reflect the company’s innovation, production, and sales in the information in real time. Commercial banks have a limited effect on reducing information asymmetry through digital transformation, and they may still be reluctant to lend for the innovation of companies. Therefore, in cities with low digital economy development, the ‘technology effect’ is difficult to enable, and the ‘technology impact’ of DTCB is smaller than the ‘scale effect’, thus increasing air pollution.

In conclusion, the ‘technology impact’ of DTCB is stronger than the ‘scale effect’ in cities with high digital economy development, reducing air pollution. In contrast, in cities with low digital economy development, the ‘technology effect’ of DTCB is smaller than the ‘scale effect’, thus increasing air pollution. Therefore, the following hypothesis is proposed.

**Hypothesis** **3.** *In cities with high digital economy development, DTCB reduces air pollution. In cities with low digital economy development, DTCB increases air pollution*.

## 3. Methodology

### 3.1. Empirical Model

Tobler’s First Law emphasizes that everything affects everything else, and the closer things are to each other, the more they affect each other [31]. The implication of the theory is that neglecting spatial correlations in an econometric analysis may lead to a biased estimator [32]. As a result, we used a spatial econometric model to examine the link between DTCB and air pollution. Referring to Lesage and pace (2009), the following model was established [33]:

(1) $$\mathrm{PM}2.5_{\mathrm{it}}=\alpha +\rho \sum_{j}w_{\mathrm{ij}}\mathrm{PM}2.5_{\mathrm{it}}+\beta_{1}{\mathrm{DTCB}}_{\mathrm{it}}+\lambda_{1}\sum_{j}w_{\mathrm{ij}}{\mathrm{DTCB}}_{\mathrm{it}}+{\gamma \mathrm{Control}}_{\mathrm{it}}+\varphi \sum_{j}w_{\mathrm{ij}}{\mathrm{Control}}_{\mathrm{it}}+\upsilon_{t}+\mu_{i}+\varepsilon_{\mathrm{it}}$$

where subscripts i and t refer to cities and years; variable DTCB denotes the degree of digital transformation of commercial banks; PM2.5 refers to levels of air pollution; spatial weight matrix is denoted by W; Control represents the control variables; the fixed effect of the city is indicated by μ; υ shows the fixed effect; ε represents the random error term; α, ρ, β, λ, γ, and φ are the parameters to be estimated.

### 3.2. Variables

(1) The explained variable: air pollution (PM2.5). Following Zhang et al. (2022) [34], we use the logarithmic values of PM2.5 concentration to measure PM2.5. Since PM2.5 concentration is the most hazardous air pollutant for people’s health, we chose each region’s annual average PM2.5 concentration to measure air pollution, expressed as PM2.5.
(2) The core explanatory variable: digital transformation of commercial banks (DTCB). This study follows the methods of studies [35,36] to measure DTCB. Firstly, this article measures the degree of DTCB at the commercial bank level by the quantity of patent permission connected to DTCB. Then, the degree of DTCB at the city level is constructed based on the degree of DTCB at the commercial bank level and the geographic distribution data of the commercial bank branches.
(3) Control variables

Considering that meteorological factors can affect the concentration of PM2.5, the average rainfall (Rain), wind velocity (Wind), and intensity of sunshine (Sun) of the cities are selected. Annual data from the above meteorological factors are used as the first type of control variable. Considering that economic factors may affect PM2.5 concentration [37,38,39], per capita GDP (GDP), foreign direct investment (FDI), industrial structure (Indus), technological innovation (Tech), and population density (Pop) are selected as the second type of control variable. Per capita GDP and population density are logarithms. To determine the industrial structure, we utilize the ratio of tertiary industry added value to secondary industry added value. We use the logarithm of the number of patents granted to a city to gauge technological innovation.

(4) Weight matrix

Following You and Lv (2018) and Nan et al. (2022) [40,41], the spatial weight matrix is built using the inverse Euclidean distance between a city and its adjacent cities. In addition, we use other spatial weight matrices for robustness testing.

### 3.3. Data Sources

The study uses a sample of 298 Chinese cities from 2010–2020. PM2.5 data was obtained from the China Air Quality Online Monitoring and Analysis Platform. Data on meteorological factors were taken from the National Meteorological Science Data Sharing Service Platform. The data of other control variables were derived from the China Statistical Yearbook. In addition, the missing data were filled by the interpolation method. Table 1 reports the descriptive statistics of the variables.

## 4. Results

### 4.1. Spatial Correlation Test

**Figure 2:**
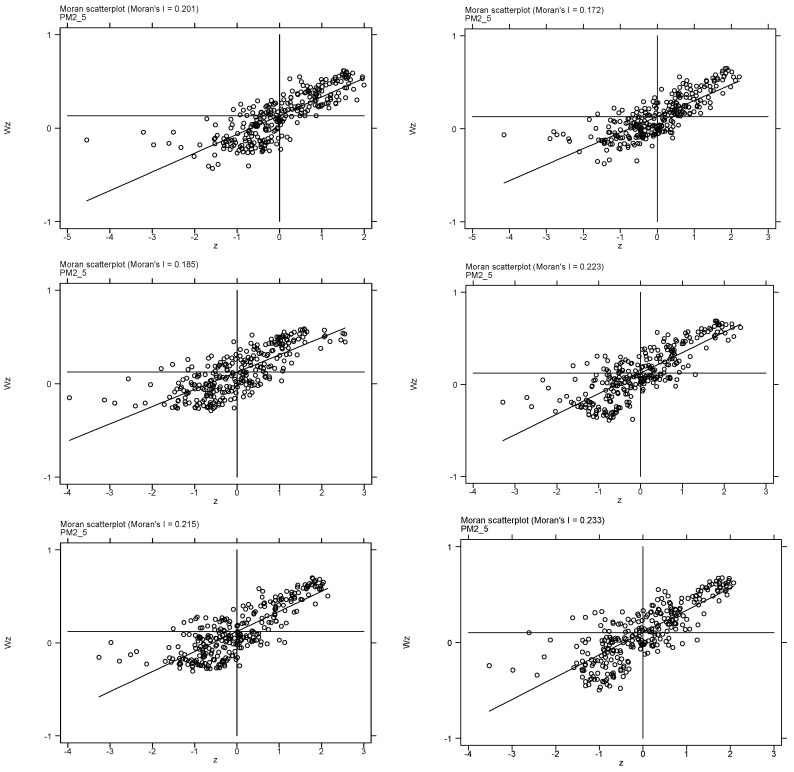
Scatter plots of the local Moran’s index of air pollution in Chinese cities.

**Figure 3:**
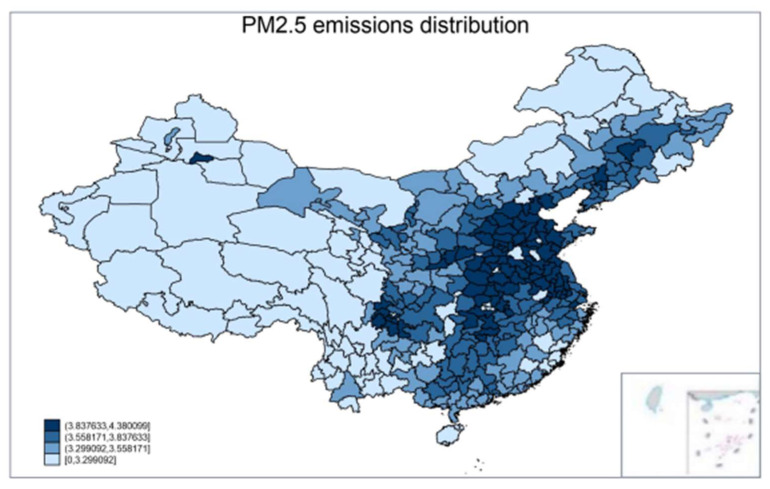
Air pollution distribution. Note: The arithmetic mean value of air pollution (2010–2020) is adopted.

The spatial correlation of air pollution was identified using Moran’s I index. Table 2 shows the test results of Moran’s I for each year (2010, 2012, 2014, 2016, 2018, 2020). It can be seen that the air pollution Moran’s I index is significantly over zero, which means that the spatial dependence of air pollution is significantly positive. In addition, Figure 2 shows Moran’s I scatter plots. It can be seen that the points are mainly concentrated in quadrants I and III, which means that air pollution levels in adjacent regions are similar. Air pollution distribution is also shown in the Figure 3.

### 4.2. The Baseline Results

Before estimation, the optimal spatial econometric model must be selected [42,43,44,45,46,47,48,49]. Hence, we test whether the spatial Durbin model (SDM) can be simplified into a spatial lag model or a spatial error model. Table 3 reports the test results. These two hypotheses are rejected, which means that the SDM is optimal.

Table 4 shows the SDM results, which shows that the direct effect of DTCB is insignificant, indicating that DTCB has not significantly increased local air pollution. The indirect effect of DTCB is significantly positive, which means that DTCB significantly increases air pollution in surrounding cities. ρ is significantly positive, indicating an apparent spatial dependence on air pollution. This indicates that local air pollution increases air pollution in surrounding cities.

Although the SDM regression provides some results, the coefficient of DTCB cannot accurately reflect the marginal effect of DTCB on air pollution. Therefore, the direct and indirect effects of DTCB are presented in Table 5. The direct effect comprises two parts: the direct influence of DTCB on local air pollution and the feedback effect. That is, DTCB affects air pollution in the neighboring city and then affects local air pollution in turn. In Table 5, the direct, indirect, and total effects of DTCB are all significantly positive, indicating that DTCB increases air pollution in local and surrounding cities. The possible reason for this is that the ‘scale effect’ of DTCB is more significant than its ‘technology effect’, so DTCB increases air pollution. Thus, Hypothesis 1a is verified. In addition, it should be noted that the direct effect of DTCB is much smaller than the spillover effect. The primary causes for this are as follows: first, the spillover effect refers to the sum of the spillover effect to all surrounding cities; second, pollutant emissions can quickly diffuse to neighboring cities.

Rain, Wind, and Sun all have substantial negative effects, suggesting that the more rainfall, the greater the wind speed. The longer the duration of sunshine, the lower the concentration of PM2.5. The direct effect of Tech, Indus, and Pop is significantly negative, which means that these factors have a significantly negative impact on air pollution. The direct effect of FDI is insignificant, indicating that FDI cannot reduce air pollution.

### 4.3. The Robustness Test

Next, we use the following methods for robustness testing: firstly, we change the measurement method of DTCB; secondly, we change the weight matrix; finally, we use the lag value of DTCB and PM2.5, respectively, to solve possible endogenous problems.

#### 4.3.1. Replace the Measurement Method of DTCB

Considering that different DTCB measurement methods may affect the estimation results, we changed the DTCB measurement methods to conduct a robustness test. This paper refers to the method of Zhang et al. (2022) to construct the indicator of DTCB [50], including the following four steps: (1) The five most widely used digital technologies in commercial banks are selected as keywords, which are big data, artificial intelligence, cloud computing, blockchain, and the Internet of Things. (2) Web crawl technology is used to obtain news search results for the combination of commercial bank names and keywords (such as ‘construction bank’ + ‘big data’) each year and the total number of news search results is calculated for the combination of commercial banks and each keyword in each year from 2010 to 2020. (3) The logarithm is taken of the total number of news search results as the level of digital transformation of a commercial bank. (4) The degree of DTCB at the city level is constructed based on the degree of DTCB at the commercial bank level and the geographic distribution data of the commercial bank branches (as shown in Table 6). The study findings are in line with the benchmark regression, showing that our conclusion is reliable.

#### 4.3.2. Use Different Spatial Weight Matrix

Referring to Corrado and Fingleton (2012) [51], this paper employs the matrix closest to the k neighbor for a robustness check. Table 7 reports the regression results. The results are still robust, which supports the core conclusion of this paper.

#### 4.3.3. Treatment of Endogeneity with the First-Order Lagged Value of DTCB

Referring to the approach of Meng and Huang (2018), to address the endogeneity issue, we employ the first-order lagged value of DTCB as a proxy variable for DTCB [52]. Table 8 reports the estimated results. It can be shown that L.DTCB’s direct and indirect effects are still good, which confirms our findings.

#### 4.3.4. Treatment of Endogeneity with the First-Order Lagged Value of PM2.5

Air pollution is often sequentially correlated, which means that the air pollution of the previous period will affect the air pollution of the current period. Therefore, we introduced the first-order lag value of PM2.5 into the model to alleviate the endogeneity problems induced by missing variables. Table 9 reports the estimated results. The results remain unchanged, again confirming our conclusion.

### 4.4. Heterogeneity Test

#### 4.4.1. Heterogeneity of Innovation Ability

The following model is adopted to test whether the impact of DTCB on air pollution is significantly different in cities with varying innovation capabilities:

(2) $$\mathrm{PM}2.5_{\mathrm{it}}=\alpha +\rho \sum_{j}w_{\mathrm{ij}}\mathrm{PM}2.5_{\mathrm{it}}+\beta_{1}{\mathrm{DTCB}}_{\mathrm{it}}+\lambda_{1}\sum_{j}w_{\mathrm{ij}}{\mathrm{DTCB}}_{\mathrm{it}}+\beta_{2}{\mathrm{DTCB}}_{\mathrm{it}}\times {\mathrm{Innovation}}_{\mathrm{it}}+\;\lambda_{2}\sum_{j}w_{\mathrm{ij}}{\mathrm{DTCB}}_{\mathrm{it}}\times {\mathrm{Innovation}}_{\mathrm{it}}+{\gamma \mathrm{Control}}_{\mathrm{it}}+\varphi \sum_{j}w_{\mathrm{ij}}{\mathrm{Control}}_{\mathrm{it}}+\upsilon_{t}+\mu_{i}+\varepsilon_{\mathrm{it}}$$

where innovation denotes innovation ability, innovation ability is a dummy variable, innovative cities are set to 1, and non-innovative cities are set to 0. The list of innovative national cities is the criterion to determine whether a city is an innovative city or not. The specific approach is as follows. Because the list of innovative cities was published since 2007, the empirical test of this paper uses data up to 2020. Therefore, cities that were included in the list of National Innovative Cities from 2007 to 2020 are considered innovative cities, while cities that have never been included in the list by 2020 are considered non-innovative cities. DTCB*Innovation is the interaction term between DTCB and innovation ability.${\;\beta }_{1}$ and $\beta_{1}+\beta_{2}$ reflect the impact of DTCB on local air pollution in non-innovative and innovative cities, respectively. $\lambda_{1}$ and $\lambda_{1}+\lambda_{2}$ reflect the spatial effects of DTCB on air pollution in non-innovative and innovative cities, respectively. The meaning of other variables and parameters refers to the settings of Equation (1).

Table 10 reports the results of the heterogeneity test. The coefficients of DTCB in columns (1) and columns (2) are significantly positive, indicating that, in non-innovative cities, DTCB increases air pollution in local and surrounding cities. The coefficients of DTCB and DTCB*Innovation in column (1) are both significant, and their sum is −0.03, indicating that, in innovative cities, DTCB reduces the air pollution of local cities. The coefficients of DTCB and DTCB*Innovation in column (2) are both significant, and their sum is −6.901, indicating that, in non-innovative cities, DTCB decreases the air pollution of surrounding cities. In conclusion, DTCB increases air pollution in local and neighboring cities in non-innovative cities. However, DTCB reduces the M2.5 emissions of local and surrounding cities in innovative cities. This verifies Hypothesis 2, and DTCB can effectively promote technological innovation in innovative cities by easing financing constraints, thus reducing local air pollution. Due to the spatial spillover effect of technology, DTCB promotes technological innovation in surrounding cities by significantly promoting local technological innovation, thus reducing air pollution in surrounding cities. In non-innovative cities, although DTCB has increased lending to enterprises, it has not promoted technological innovation. In other words, DTCB has only promoted economic development and has not propelled technological innovation. Therefore, DTCB increases air pollution in local and surrounding cities by promoting the economic development of the local and surrounding cities.

#### 4.4.2. Heterogeneity of the Degree of Development of the Digital Economy

The following model is adopted to test whether the impact of DTCB on air pollution is significantly different in cities with varying degrees of digital economy development:

(3) $$\mathrm{PM}2.5_{\mathrm{it}}=\alpha +\rho \sum_{j}w_{\mathrm{ij}}\mathrm{PM}2.5_{\mathrm{it}}+\beta_{1}{\mathrm{DTCB}}_{\mathrm{it}}+\lambda_{1}\sum_{j}w_{\mathrm{ij}}{\mathrm{DTCB}}_{\mathrm{it}}+\beta_{2}{\mathrm{DTCB}}_{\mathrm{it}}\times \;{\mathrm{Digital}}_{\mathrm{it}}+\lambda_{2}\sum_{j}w_{\mathrm{ij}}{\mathrm{DTCB}}_{\mathrm{it}}\times {\mathrm{Digital}}_{\mathrm{it}}+{\gamma \mathrm{Control}}_{\mathrm{it}}+\varphi \sum_{j}w_{\mathrm{ij}}{\mathrm{Control}}_{\mathrm{it}}+\upsilon_{t}+\mu_{i}+\varepsilon_{\mathrm{it}}$$

where Digital denotes the degree of digital economy development, and is a dummy variable, and the city with high digital economy development is taken as 1, and the remaining cities are taken as 0. This paper identifies cities with high digital economy development according to the ‘China Digital Economy Industry Development Index Report 2021′ compiled by the Big Data Analysis Technology Innovation Center of Beijing Academy of Big Data. The specific approach is as follows. The 2021 China Digital Economy Industrial Development Index Report lists 20 cities with the highest level of digital economy development and divides them into three tiers. Compared with other cities, these 20 cities have a noticeably higher degree of digital economy development. Therefore, these 20 cities are included in the group with a high degree of digital economy development, and the remaining cities are all included in the group with low digital economy development. DTCB*Digital is the interaction term between DTCB and the degree of development of the digital economy.${\;\beta }_{1}$ and $\beta_{1}+\beta_{2}$ reflect the impact of DTCB on local air pollution in cities with low digital economy development and cities with high digital economy development, respectively. $\lambda_{1}$ and $\lambda_{1}+\lambda_{2}$ reflect the spatial effects of DTCB on air pollution in cities with low digital economy development and cities with high digital economy development. The meaning of other variables and parameters refers to the settings of Equation (1).

Table 11 reports the results of the heterogeneity test. The coefficients of DTCB in columns (1) and (2) are significantly positive, indicating that in cities with low digital economy development, DTCB increases air pollution in local and surrounding cities. The coefficients of DTCB and DTCB*Digital in column (1) are both significant, and their sum is −0.085, indicating that, in cities with high digital economy development, DTCB reduces air pollution in local cities. The coefficients of DTCB and DTCB*Digital in column (2) are both significant, and their sum is −17.72, indicating that, in cities with high digital economy development, DTCB reduces the air pollution of surrounding cities. The above results verify Hypothesis 3. The possible reason for this is that, in cities with high development of the digital economy, DTCB can better solve the problem of information asymmetry, ease the financing constraints of the technological innovation of companies, and ultimately reduce air pollution. Since the spatial spillover effect of technology is positive, DTCB promotes technological innovation in the surrounding cities, thereby reducing air pollution in the surrounding cities. In contrast, in cities with low digital economy development, DTCB only promotes economic development and does not propel technological innovation. Therefore, DTCB increases air pollution in local and surrounding cities by promoting the economic development of the local and surrounding cities.

### 4.5. Mechanism Analysis

Theoretical analysis shows that DTCB may affect air pollution by exerting a ‘technology effect’ and a ‘scale effect’. Hence, the following model is adopted to examine the effect of DTCB on economic development and technological innovation:

(4) $${\mathrm{Med}}_{\mathrm{it}}=\alpha +\rho \sum_{j}w_{\mathrm{ij}}{\mathrm{Med}}_{\mathrm{it}}+\beta_{1}{\mathrm{DTCB}}_{\mathrm{it}}+\lambda_{1}\sum_{j}w_{\mathrm{ij}}{\mathrm{DTCB}}_{\mathrm{it}}+{\gamma \mathrm{Control}}_{\mathrm{it}}+\varphi \sum_{j}w_{\mathrm{ij}}{\mathrm{Control}}_{\mathrm{it}}+\upsilon_{t}+\mu_{i}+\varepsilon_{\mathrm{it}}$$

where Med denotes the intermediary variable, representing economic growth and technological innovation, and other variables have the same meaning as Equation (1). Table 12 reports the estimated results.

The coefficient of DTCB in column (1) and column (2) is significantly positive, indicating that DTCB significantly promotes the economic growth of local and surrounding cities. The possible reason for this is that DTCB increases the supply of loans to enterprises. It encourages enterprises to expand scale or launch new products, thus promoting economic growth at the macro level. As the spatial correlation of economic development is positive, DTCB promotes the economic development of neighboring cities by promoting local economic growth.

The coefficients of DTCB in column (4) and column (5) are insignificant, indicating that DTCB cannot promote technological innovation in local and surrounding cities. The possible reason for this is that, although DTCB increases the supply of loans to enterprises, instead of investing in technological innovation due to their low innovation capacity, these companies expand the scale of production. Since the DTCB cannot promote technological innovation in local cities, it cannot promote technological innovation in neighboring cities through the spatial effects of technology innovation.

In short, DTCB increases local air pollution by promoting local economic growth. This is because DTCB can significantly promote local economic growth, but cannot propel local technological innovation. DTCB increases the air pollution of surrounding cities through the following two paths. First, there is a spatial correlation between PM2.5 in local and surrounding areas, making DTCB increase air pollution in surrounding cities by increasing local air pollution. Second, the spatial correlation of economic development is positive, which makes DTCB promote the economic development of surrounding cities by promoting local economic development, thus increasing the air pollution of surrounding cities.

## 5. Conclusions

Economic activities are an important cause of air pollution. DTCB has increased the supply of loans, which can affect air pollution by promoting economic development. However, few scholars have studied the spatial effects of DTCB on air pollution. This research uses a spatial Durbin model to investigate the spatial impacts of DTCB on air pollution using city-level data from 2010 to 2020. The empirical results show that DTCB significantly increases air pollution in local and surrounding cities. Heterogeneity analysis shows that, in non-innovative cities and cities with low digital economy development, DTCB significantly increases air pollution. In contrast, in innovative cities and cities with high digital economy development, DTCB significantly reduces air pollution. Mechanism analysis shows that DTCB has no significant impact on technological innovation, but significantly promotes economic development. Therefore, DTCB increases air pollution by promoting economic growth.

Based on the above conclusions, the following implications can be derived from our study. The government should be aware of the negative impact of DTCB on air pollution. This paper confirms that the main reason why DTCB increases air pollution is that it only promotes economic growth but does not promote technological innovation. Therefore, the government should guide commercial banks to apply digital technologies to increase lending for technological innovation and thus reduce air pollution. In innovative cities and cities with high development of the digital economy, DTCB can promote technological innovation, thus reducing air pollution. Therefore, the government should improve the innovation capacity of cities and promote the development of the digital economy in cities so that DTCB can promote technological innovation to reduce PM2.5 emissions.

## Figures and Tables

**Table 1:** Descriptive statistics.

Variables	Obs	Mean	Std. Dev.	Min	Max
PM2.5	3157	3.687	0.354	2.241	4.687
DTCB	3157	0.733	0.476	0	1.683
Rain	3157	6.855	0.477	5.217	7.917
Wind	3157	0.744	0.238	−0.030	1.515
Sun	3157	7.556	0.269	6.609	8.128
Pop	3157	5.733	0.927	1.609	7.882
FDI	3157	0.003	0.00270	0.001	0.030
Indus	3157	1.007	0.570	0.109	5.348
Tech	3157	7.176	1.773	0.693	12.31
GDP	3157	5.830	0.553	4.103	8.345

**Table 2:** Statistical tests of spatial autocorrelation using Moran’s I.

	2010	2012	2014	2016	2018	2020
PM2.5	0.201 ***	0.172 ***	0.185 ***	0.223 ***	0.215 ***	0.233 ***

Notes: *** denotes significance of 1%.

**Table 3:** Model selection.

	$\chi^{2}$	*p*-Value
SDM versus SLM	310.32	0.0000
SDM versus SEM	368.14	0.0000

**Table 4:** Result of spatial Durbin models.

Variables	Coefficient	Std. Err
DTCB	0.006	(1.34)
Rain	0.010	(0.48)
Wind	−0.065 ***	(−2.63)
Sun	−0.131 ***	(−3.64)
Pop	−0.041	(−0.80)
FDI	−2.196 **	(−2.17)
Indus	−0.012	(−1.00)
W*DTCB	0.143 **	(2.17)
W*Rain	−0.357 ***	(−4.60)
W*Wind	−0.944 ***	(−5.29)
W*Sun	0.326 ***	(2.62)
W*Pop	−1.999 ***	(−4.35)
W*FDI	5.146	(0.62)
W*Indus	0.272 ***	(3.46)
ρ	0.977 ***	(699.19)
*N*	3157	
*R* ^2^	0.2951	

Notes: *** and ** denote significance of 1% and 5% respectively. Numbers in () represent standard errors.

**Table 5:** Effects of spatial Durbin models for values in Table 4.

Variables	Direct Effect		Indirect Effect		Total Effect	
	Coefficients	Std. Err	Coefficients	Std. Err	Coefficients	Std. Err
DTCB	0.028 **	(2.42)	6.313 **	(2.22)	6.341 **	(2.22)
Rain	−0.045 ***	(−3.30)	−15.021 ***	(−5.56)	−15.066 ***	(−5.59)
Wind	−0.222 ***	(−7.93)	−44.637 ***	(−5.96)	−44.860 ***	(−5.97)
Sun	−0.099 ***	(−3.68)	−8.910 **	(−2.17)	−8.811 **	(−2.15)
Pop	−0.363 ***	(−5.86)	−89.493 ***	(−5.04)	−89.856 ***	(−5.04)
FDI	−1.598	(−1.03)	142.157	(0.39)	140.558	(0.39)
Indus	0.029 *	(1.76)	11.334 ***	(3.34)	11.363 ***	(3.34)

Notes: ***, ** and * denote significance of 1%, 5%, and 10%, respectively. Numbers in () represent standard errors.

**Table 6:** Robust test of replacing core explanatory variables.

Variables	Direct Effects		Spillover Effects		Total Effects	
	Coefficients	Std. Err	Coefficients	Std. Err	Coefficients	Std. Err
DTCB	0.007 *	(1.75)	0.166 *	(1.84)	0.173 *	(1.93)
Rain	−0.027	(−1.20)	−0.139	(−1.33)	−0.166 *	(−1.70)
Wind	−0.067 ***	(−3.18)	−0.561	(−1.59)	−0.628 *	(−1.73)
Sun	−0.042	(−1.17)	−0.101	(−0.51)	−0.143	(−0.75)
Pop	−0.030	(−0.67)	−0.853	(−0.77)	−0.883	(−0.78)
FDI	−2.423 ***	(−2.64)	−7.300	(−0.48)	−9.723	(−0.61)
Indus	−0.001	(−0.07)	0.149	(0.87)	0.148	(0.82)

Notes: *** and * denote significance of 1% and 10%, respectively. Numbers in () represent standard errors.

**Table 7:** Robust test using different weight matrix.

Variables	Direct Effects		Spillover Effects		Total Effects	
	Coefficients	Std. Err	Coefficients	Std. Err	Coefficients	Std. Err
DTCB	0.006 *	(1.91)	0.488 **	(2.09)	0.493 **	(2.03)
Rain	−0.046 ***	(−4.12)	0.092	(0.69)	0.046	(0.34)
Wind	−0.163 ***	(−6.99)	0.366	(0.36)	0.203	(0.20)
Sun	−0.131 ***	(−5.68)	−0.584 ***	(−2.58)	−0.715 ***	(−3.10)
Pop	0.033	(0.53)	2.035	(1.51)	2.068	(1.54)
FDI	−2.990 ***	(−3.13)	35.855	(0.77)	32.865	(0.71)
Indus	−0.021 *	(−1.73)	−0.592	(−1.10)	−0.613	(−1.14)

Notes: ***, ** and * denote significance of 1%, 5% and 10%, respectively. Numbers in () represent standard errors.

**Table 8:** Robust test with the first order lagged value of Fintech.

Variables	Direct Effects		Spillover Effects		Total EFFECTs	
	Coefficients	Std. Err	Coefficients	Std. Err	Coefficients	Std. Err
L.DTCB	0.029 **	(2.39)	8.121 ***	(2.91)	8.150 ***	(2.91)
Rain	−0.038 ***	(−2.74)	−12.732 ***	(−4.65)	−12.770 ***	(−4.67)
Wind	−0.256 ***	(−9.54)	−54.713 ***	(−7.75)	−54.970 ***	(−7.77)
Sun	−0.071 ***	(−2.66)	9.651 **	(2.27)	9.581 **	(2.26)
Pop	−0.620 ***	(−8.47)	−148.739 ***	(−7.85)	−149.360 ***	(−7.86)
FDI	−0.768	(−0.44)	480.467	(1.14)	479.698	(1.14)
Indus	0.024	(1.44)	10.734 ***	(3.35)	10.757 ***	(3.35)

Notes: *** and ** denote significance of 1% and 5% respectively. Numbers in () represent standard errors.

**Table 9:** The robustness testing of the first order lag term of PM2.5 is introduced into the explanatory variables.

Variables	Direct Effects		Spillover Effects		Total Effects	
	Coefficients	Std. Err	Coefficients	Std. Err	Coefficients	Std. Err
L.PM2.5	0.278 ***	(7.99)	0.088 **	(2.08)	0.366 ***	(7.04)
DTCB	0.009 *	(1.74)	0.053 *	(1.86)	0.061 *	(1.78)
Rain	−0.012	(−0.78)	0.004	(0.16)	−0.008	(−0.27)
Wind	−0.058 ***	(−3.01)	0.168 *	(1.95)	0.110	(1.30)
Sun	−0.076 ***	(−2.70)	0.039	(0.85)	−0.037	(−0.66)
Pop	−0.020	(−0.43)	0.078	(0.34)	0.058	(0.27)
FDI	−2.394 ***	(−2.67)	−0.099	(−0.02)	−2.493	(−0.58)
Indus	−0.009	(−0.95)	−0.014	(−0.36)	−0.024	(−0.66)

Notes: ***, ** and * denote significance of 1%, 5% and 10%, respectively. Numbers in () represent standard errors.

**Table 10:** Heterogeneity of Innovation Ability.

Variables	Direct Effects		Spillover Effects		Total Effects	
	(1)		(2)		(3)	
	Coefficients	Std. Err	Coefficients	Std. Err	Coefficients	Std. Err
DTCB	0.036 ***	(2.88)	8.093 ***	(2.58)	8.129 ***	(2.58)
DTCB*Digital	−0.121 ***	(−3.76)	−25.813 ***	(−3.57)	−25.934 ***	(−3.57)
Rain	−0.043 ***	(−3.19)	−16.980 ***	(−6.34)	−17.024 ***	(−6.37)
Wind	−0.241 ***	(−8.75)	−49.070 ***	(−6.65)	−49.310 ***	(−6.67)
Sun	−0.098 ***	(−3.63)	6.240	(1.53)	6.142	(1.51)
Pop	−0.317 ***	(−4.89)	−81.028 ***	(−4.33)	−81.345 ***	(−4.34)
FDI	−2.895 **	(−1.96)	−157.164	(−0.44)	−160.059	(−0.45)
Indus	0.023	(1.35)	10.154 ***	(3.08)	10.177 ***	(3.08)

Notes: *** and ** denote significance of 1% and 5%. Numbers in () represent standard errors.

**Table 11:** Heterogeneity of the Degree of Development of the Digital Economy.

Variables	Direct Effects		Spillover Effects		Total Effects	
	(1)		(2)		(3)	
	Coefficients	Std. Err	Coefficients	Std. Err	Coefficients	Std. Err
DTCB	0.045 ***	(3.59)	9.895 ***	(3.08)	9.939 ***	(3.09)
DTCB*Innovation	−0.075 ***	(−4.18)	−16.796 ***	(−3.73)	−16.870 ***	(−3.73)
Rain	−0.047 ***	(−3.54)	−17.608 ***	(−6.39)	−17.655 ***	(−6.42)
Wind	−0.258 ***	(−8.84)	−54.475 ***	(−7.06)	−54.734 ***	(−7.07)
Sun	−0.105 ***	(−3.84)	4.318	(1.05)	4.213	(1.02)
Pop	−0.425 ***	(−6.81)	−105.759 ***	(−5.96)	−106.184 ***	(−5.97)
FDI	−2.200	(−1.49)	15.324	(0.04)	13.125	(0.04)
Indus	0.020	(1.21)	9.372 ***	(2.85)	9.392 ***	(2.84)

Notes: *** denotes significance of 1%. Numbers in () represent standard errors.

**Table 12:** Mechanism test.

Variables	GDP			Tech		
	Direct Effects	Spillover Effects	Total Effects	Direct Effects	Spillover Effects	Total Effects
	(1)	(2)		(3)	(4)	
DTCB	0.046 ***	8.489 ***	8.535 ***	−0.049	−0.713	−0.761
	(3.18)	(2.91)	(2.91)	(−1.15)	(−0.44)	(−0.46)
Pop	0.008	−24.774	−24.766	0.735	30.239	30.974
	(0.04)	(−1.13)	(−1.13)	(1.18)	(1.35)	(1.38)
FDI	11.763 ***	2267.869 ***	2279.632 ***	7.892	184.438	192.330
	(4.48)	(4.03)	(4.04)	(0.76)	(0.73)	(0.76)
Indus	−0.171 ***	−17.670 ***	−17.841 ***	−0.021	1.514	1.493
	(−7.28)	(−4.18)	(−4.21)	(−0.19)	(0.68)	(0.68)

Notes: *** denotes significance of 1%.

## Data Availability

Not applicable.
